# *Coxiella burnetii* endocarditis – a real threat in the context of Q fever re-emergence

**DOI:** 10.1186/1471-2334-14-S7-P32

**Published:** 2014-10-15

**Authors:** Anca-Ruxandra Negru, Cristina Popescu, Mihaela Rădulescu, Alina Lobodan, Irina Lăpădat, Smaranda Gliga, Raluca Dulamă, Doina Cristea, Victoria Aramă

**Affiliations:** 1 National Institute for Infectious Diseases "Prof. Dr. Matei Balş", Bucharest, Romania; 2 Carol Davila University of Medicine and Pharmacy, Bucharest, Romania

## Background

Q fever is a disease with worldwide distribution. The real number of patients with this disease is underestimated due to its nonspecific symptoms and because of the difficulties associated with serological diagnosis. Some of the patients diagnosed in the first stage with Q fever later develop chronic disease. Endocarditis is the most frequent and severe form of chronic Q fever. In the National Institute for Infectious Diseases "Prof. Dr. Matei Balş" the number of patients diagnosed with Q fever has significantly increased in the last few years. The number of cases from the first half of 2014 almost exceeded the whole number recorded in 2013. In these circumstances we expect that the number of cases of endocarditis with *Coxiella burnetii* will rise.

## Methods

We retrospectively analyzed the patients diagnosed with Q fever with endocarditis in the Matei Balş Institute between 2011 and 2014. Of 89 patients with Q fever, 4 patients met the inclusion criteria (endocarditis plus serologic diagnosis of *Coxiella burnetii*).

## Results

All the patients were males with a median age of 58.25 years. All of them lived in urban area and were admitted for prolonged fever. Three out of four patients presented hepatitis with ALT elevation and cholestasis at admission. All blood cultures taken during hospitalization were negative. Endocarditis diagnosis was established after echographic evaluation. In three cases the aortic valve was affected while in the other case the aortic valve was impaired. Detection of IgM antibodies for *Coxiella burnetii* by ELISA was used as screening test for the etiologic diagnostic that was confirmed by dosing phase I and II IgM and IgG antibodies. All patients initially received empiric therapy with association between betalactams and aminoglycosides. After the etiologic diagnosis was established, the patients were treated with doxycycline in association with ofloxacin/rifampin.

## Conclusion

*Coxiella burnetii* must be considered as a possible etiologic agent in case of endocarditis with negative blood cultures especially in the context of increasing numbers of patients diagnosed with Q fever. The association between fever, hepatitis and interstitial pneumonia must be an additional argument for considering chronic Q fever with endocardial impairment a possible diagnosis.

